# Conductance-photoacoustic spectroscopy for fast and concurrent sensing of hydrogen and hydrocarbons

**DOI:** 10.1016/j.pacs.2025.100752

**Published:** 2025-07-23

**Authors:** Ruobin Zhuang, Jianfeng He, Haoyang Lin, Huijian Luo, Leqing Lin, Lihao Wang, Bin Liu, Wenguo Zhu, Yongchun Zhong, Jianhui Yu, Markus Sigrist, Huadan Zheng

**Affiliations:** a International Cooperation Joint Laboratory for Optoelectronic Hybrid Integrated Circuits, Jinan University, Guangzhou 510632, China; b Guangdong-Hong Kong-Macao Joint Laboratory for Intelligent Micro-Nano Optoelectronic Technology, Foshan University, Foshan 528000, China; c Physics Department, ETH Zürich, John-von-Neumann-Weg 9, Zurich CH-8093, Switzerland

**Keywords:** Photoacoustic spectroscopy, QEPAS, Quartz tuning fork, Quartz-enhanced photoacoustic spectroscopy

## Abstract

Accurate and rapid detection of hydrogen and hydrocarbons is critical for safety and efficiency in modern energy, industrial, and environmental systems. However, selective and simultaneous quantification of these species remains a significant technical challenge. Here, we introduce conductance–photoacoustic spectroscopy (ConPAS), an integrated sensing approach that combines conductance-based resonance modulation with quartz-enhanced photoacoustic spectroscopy in a single device. By bridging a quartz tuning fork (QTF) with a catalytic platinum microwire, ConPAS enables concurrent extraction of hydrogen and hydrocarbon concentrations from a unified electrical signal: hydrogen is quantified by frequency analysis, while hydrocarbon content is determined by amplitude analysis simultaneously. Experiments demonstrate minimum detection limits of 0.69 % for hydrogen, 40.26 ppm for propane, and 133.7 ppm for methane, with millisecond response time and excellent linearity (R² > 0.99). The modular architecture allows flexible adaptation to other analytes via material substitution, offering a scalable and versatile solution for simultaneous, multi-component gas sensing. This work establishes ConPAS as a powerful, calibration-compatible platform for integrated gas analysis in hydrogen-enriched environments, with broad implications for safety monitoring, process control, and advanced energy applications.

## 1 Introduction

Optical spectroscopy has long been recognized as a cornerstone methodology for gas sensing, owing to its non-invasive nature, high molecular specificity, and excellent detection sensitivity [1], [2], [3], [4], [5]. Trace gas detection and quantification have been widely applied in environmental, industrial, and biomedical contexts using a variety of spectroscopic techniques, including tunable diode laser absorption spectroscopy (TDLAS) [6], [7], cavity ring-down spectroscopy (CRDS) [8], [9], Fourier-transform infrared (FTIR) spectroscopy [10], [11], Raman spectroscopy [12], [13], and photoacoustic spectroscopy (PAS) [14], [15], [16], [17], [18], [19], [20]. These methods leverage characteristic molecular absorption or scattering features in the infrared or visible spectral domains, allowing for selective identification of target analytes even in chemically complex mixtures [21]. Among these, PAS has attracted particular interest for its ability to convert absorbed optical energy directly into acoustic waves, thereby enabling highly compact sensor architectures [16], [17], [22], [23], [24]. Unlike conventional absorption-based techniques that rely on photodetectors, PAS detects pressure waves generated by non-radiative molecular relaxation following laser excitation, which are then transduced via a microphone or piezoelectric element [25], [26], [27]. This modality enhances detection sensitivity across a broad spectral range[26], [28], [29]. A major advancement in PAS is quartz-enhanced photoacoustic spectroscopy (QEPAS), which replaces conventional microphones with a high-Q quartz tuning fork (QTF) as the acoustic transducer [30], [31], [32], [33]. The high mechanical quality factor and narrowband frequency response of the QTF facilitate efficient noise suppression and allow atmospheric operation without acoustic chambers [32], [34], [35], [36], [37]. To date, QEPAS has been successfully applied to the detection of dozens of gas species, including greenhouse gases, industrial pollutants, and biomarkers, achieving detection limits spanning from parts-per-billion to parts-per-million levels [38], [39], [40], [41], [42], [43], [44].

Hydrogen plays an indispensable role across a broad range of scientific and engineering domains, including industrial manufacturing, agricultural processes, biomedical applications, and energy systems [45], [46], [47], [48], [49]. In industrial chemistry, hydrogen serves as a fundamental feedstock in petroleum refining, ammonia synthesis, and hydrogenation reactions [50], [51]. The integration of hydrogen into energy grids, as a clean fuel, has accelerated in the context of carbon-neutral energy strategies [48], [52], [53]. Hydrogen possesses several attractive physicochemical properties, including a low ignition threshold, high diffusivity, and a wide flammability range. However, these characteristics also bring safety risks [54], [55]. Consequently, the precise and continuous monitoring of hydrogen concentration in complex environments has become a critical requirement in both applied research and practical deployment[56], [57]. In many emerging applications, particularly in hydrogen-enriched natural gas (HENG) systems, reforming processes, and fuel blending technologies, hydrogen is frequently mixed with hydrocarbons such as methane, propane, or natural gas[57], [58]. Accurate, real-time quantification of both hydrogen and hydrocarbon components is crucial not only for maintaining optimal energy content and combustion stoichiometry but also for ensuring safe operation by preventing explosive mixtures[59]. Moreover, since the flammability limits, ignition energies, and diffusion rates of hydrogen and hydrocarbons differ significantly, even small deviations in their concentrations can substantially impact system safety and performance[60], [61]. As a result, there is a growing demand for sensing platforms capable of simultaneously and selectively measuring hydrogen and hydrocarbon species within dynamic, multi-component gas environments.

Although optical spectroscopy offers numerous advantages for gas detection, its application to hydrogen sensing remains fundamentally limited by the molecular properties of hydrogen. As a homonuclear diatomic molecule without a permanent dipole moment, hydrogen exhibits only weak quadrupole-induced rovibrational transitions, resulting in extremely low absorption cross-sections in the near- and mid-infrared regions [62], [63]. As a result, conventional spectroscopic techniques such as TDLAS and FTIR are intrinsically challenged in achieving high-sensitivity hydrogen detection, particularly in complex mixtures dominated by stronger absorbers like hydrocarbons [64], [65], [66]. To overcome these difficulties, alternative transduction methods, including metal-oxide-semiconductor (MOS) sensors, thermal conductivity detectors, catalytic sensors, and electrochemical devices, have been adopted [67], [68], [69]. In mixed-gas environments such as hydrogen–methane or hydrogen–propane systems, current solutions often rely on hybrid architectures combining optical and non-optical elements [60], [61], [70], [71], [72], which introduce challenges in signal synchronization, calibration, and system complexity [56], [73], [74]. These limitations underscore the pressing need for a unified sensing framework capable of selectively and simultaneously quantifying hydrogen and hydrocarbons.

In this work, to address the aforementioned limitations, we introduce a technique termed conductance–photoacoustic spectroscopy (ConPAS), integrating the conductance and photoacoustic transduction in a single device. It was realized by bridging a quartz tuning fork (QTF) with a catalytic platinum (Pt) microwire. This hybrid structure simultaneously supports two orthogonal sensing modalities: resonance frequency shifts induced by hydrogen adsorption on the Pt surface, and quartz-enhanced photoacoustic signals generated by infrared absorption of hydrocarbon gases. Hydrogen concentration is extracted from mechanical resonance modulation due to Pt–H₂ interactions, while hydrocarbon content is determined from photoacoustic amplitude variations under wavelength-modulated laser excitation. Both signals are encoded in a single electrical output trace, enabling decoupled yet concurrent analysis of multiple gas species. Experimental validation confirms that the ConPAS platform enables simultaneous detection of hydrogen with propane and methane, achieving minimum detection limits of 0.69 % for hydrogen, 40.26 ppm for propane, and 133.7 ppm for methane. Excellent linear responses were observed for propane, methane, and hydrogen, exhibiting an R² exceeding 0.99. The system demonstrated a rapid response time within 1 ms, highlighting its suitability for real-time multi-component gas analysis.

## 2 Methods

### 2.1 Theory

**Fig. 1:**
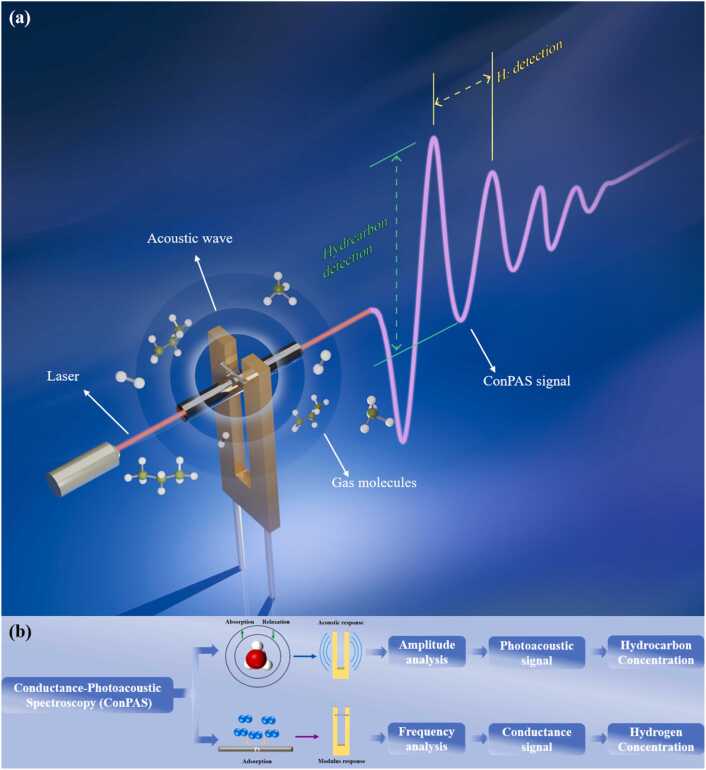
(a)Schematic illustration of the ConPAS mechanism. A platinum-bridged quartz tuning fork simultaneously responds to hydrogen-induced resonance frequency shifts and hydrocarbon-specific photoacoustic signals, enabling decoupled, real-time dual-species gas detection from a single output trace. (b) Flowchart of ConPAS system.

Fig. 1(a) illustrates the schematic diagram of the conductance–photoacoustic spectroscopy (ConPAS) system. A platinum (Pt) microwire is fabricated and bridged between the two prongs of the quartz tuning fork (QTF). Owing to the intrinsic ductility of Pt, the microwire contributes an additional elastic component to the resonator, resulting in a unified mechanical system whose overall resonance frequency is effectively modulated by the mechanical properties of the Pt microwire. When hydrogen atoms adsorb onto the surface of platinum (Pt), they occupy interstitial sites within the metal lattice. This occupation induces lattice expansion and alters the interatomic distances, which in turn leads to changes in the overall elastic modulus of the material. The change in the elastic modulus of the microwire selectively enhances the system’s sensitivity to variations in hydrogen concentration [75]. To amplify the detection of hydrocarbon species, a pair of stainless-steel micro acoustic resonator tubes is symmetrically mounted along the laser propagation axis, adjacent to the QTF prongs [76]. Each resonator has a length approximately equal to half the acoustic wavelength, with minor adjustments accounting for non-ideal half-wave resonance conditions. The QTF is positioned at the antinode of the standing acoustic wave within the resonators, with a gap of approximately 50 μm between the resonator ends and the QTF surface. This configuration ensures efficient acoustic energy coupling to the QTF, enhancing the photoacoustic signal strength for hydrocarbon absorption while maintaining the unobstructed mechanical vibration of the fork [77].

The conductance–photoacoustic spectroscopy (ConPAS) system enables simultaneous detection of hydrogen and hydrocarbon gases through a single time-resolved measurement. As shown in Fig. 1(b), by applying a sinusoidal laser modulation slightly detuned from the QTF resonance, a photoacoustic signal is generated, whose periodicity encodes the resonance frequency shift induced by hydrogen adsorption. Simultaneously, a pulsed current sweeping the laser wavelength across the hydrocarbon absorption line modulates the photoacoustic signal amplitude, where the differential between successive beat peaks and troughs correlates with hydrocarbon concentration. Owing to the independent nature of frequency and amplitude modulation, ConPAS allows decoupled, real-time, and simultaneous quantification of multiple gas species within a single QTF.

To elucidate the relationship between hydrogen concentration and the resonance frequency shift observed in the ConPAS sensor, a theoretical model based on elastic modulus modulation is developed. The resonance frequency *f* of the quartz tuning fork (QTF) system is governed by [78], [79], [80]:

(1) $$f=\frac{1}{2\pi }\times \sqrt{\frac{k_{\mathrm{sys}}}{m_{\mathrm{sys}}}}$$

where $k_{\mathrm{sys}}$ is the effective spring constant of the combined QTF and Pt microwire structure, and $m_{\mathrm{sys}}$ represents the effective mass. As hydrogen adsorption primarily modifies the elastic properties of the Pt microwire without significantly altering the system mass, the frequency shift is attributed to changes in $k_{\mathrm{sys}}$.

The system spring constant can be expressed as:

(2) $$k_{\mathrm{sys}}=k_{\mathrm{QTF}}+k_{\mathrm{pt}}$$

where $k_{\mathrm{QTF}}$ is the intrinsic spring constant of the QTF, and $k_{\mathrm{pt}}$ represents the contribution from the Pt microwire. The elastic modulus of the Pt microwire varies with hydrogen concentration *n*_*1*_, which can be modeled as:

(3) $$E_{\mathrm{Pt}}(x)=E_{\mathrm{Pt},0}+\beta n_{1}$$

where $E_{\mathrm{Pt},0}$ is the initial elastic modulus of Pt in a hydrogen-free environment, and *β* is a proportionality constant characterizing the sensitivity of the modulus to hydrogen adsorption. Accordingly, the spring constant of the Pt microwire becomes:

(4) $$k_{\mathrm{Pt}}\left(x\right)=C\left(E_{\mathrm{Pt},0}+\beta n_{1}\right)$$

where *C* is a geometric factor determined by the dimensions and boundary conditions of the microwire. Substituting into the resonance frequency expression yields:

(5) $$f=\frac{1}{2\pi }\times \sqrt{\frac{k_{\mathrm{QTF}}+C(E_{\mathrm{Pt},0}+\beta n_{1})}{m_{\mathrm{sys}}}}$$

Given that the hydrogen-induced perturbations are relatively small, a first-order Taylor expansion around $n_{1}=0$ can be applied, leading to an approximately linear dependence between frequency and hydrogen concentration:

(6) $$f\approx f_{0}+\gamma n_{1}$$

where *f*_*0*_ is the resonance frequency in the absence of hydrogen, and *γ* is an empirical constant related to *β*, *C*, and $m_{\mathrm{sys}}$.

In contrast to conventional QEPAS, the beat-frequency QEPAS (BF-QEPAS) configuration offers the capability to self-reference the resonance frequency *f*_1_ of the QTF without external frequency scanning [81], [82], [83]. Based on beat-frequency theory, the resonance frequency can be determined from the temporal spacing *Δt* between successive extrema, peaks, or troughs, in the beat signal envelope. Specifically, the frequency offset |*f*_1_ − *f*| between the QTF resonance and the applied laser modulation frequency *f* is given by:

(7) $$\left|f_{1}-f\right|=\frac{1}{∆t}$$

where *Δt* is the time interval extracted from the beat pattern. Given that the laser modulation frequency *f*_1_ is known, and *Δt* can be readily extracted from the temporal domain signal, the resonant frequency *f* can be precisely calculated. This time-domain approach eliminates the need for frequency-domain sweeping and enables in situ calibration of the QTF during measurement.

The magnitude of the beat envelope is primarily determined by the concentration of the target gas species, which governs the amount of absorbed optical power and subsequent acoustic pressure generation. To quantitatively relate the BF-QEPAS signal to gas concentration, the amplitude of the first beat maximum *A*_*1*_ and the immediately following minimum *A*_*2*_ are considered. The differential signal ∆A, defined as:

(8) $$∆A=A_{1}-A_{2}=Kn_{2}$$

where *n*_2_ is the target gas concentration, *K* is a constant determined by system parameters. Based on the combined effects of conductance spectroscopy and photoacoustic spectroscopy, the ConPAS signal *S* can be expressed as follows:

(9) $$S(t)=Ae^{-\zeta \mathrm{tsin}(2\pi \mathrm{ft})}$$

where ζ denotes the damping coefficient. Combining (7), (8), (9), the resulting system of Eq. (10) is obtained, which describes how the concentrations of hydrogen and hydrocarbon gases can be simultaneously determined from the two dimensions of the ConPAS signal.

(10) $$\left\{\begin{array}{c} \left|f_{1}-f\right|=\frac{1}{∆t}\propto n_{1}\\ ∆A=A_{1}-A_{2}\propto n_{2} \end{array}\right)$$

### 2.2 Experimental

**Fig. 2:**
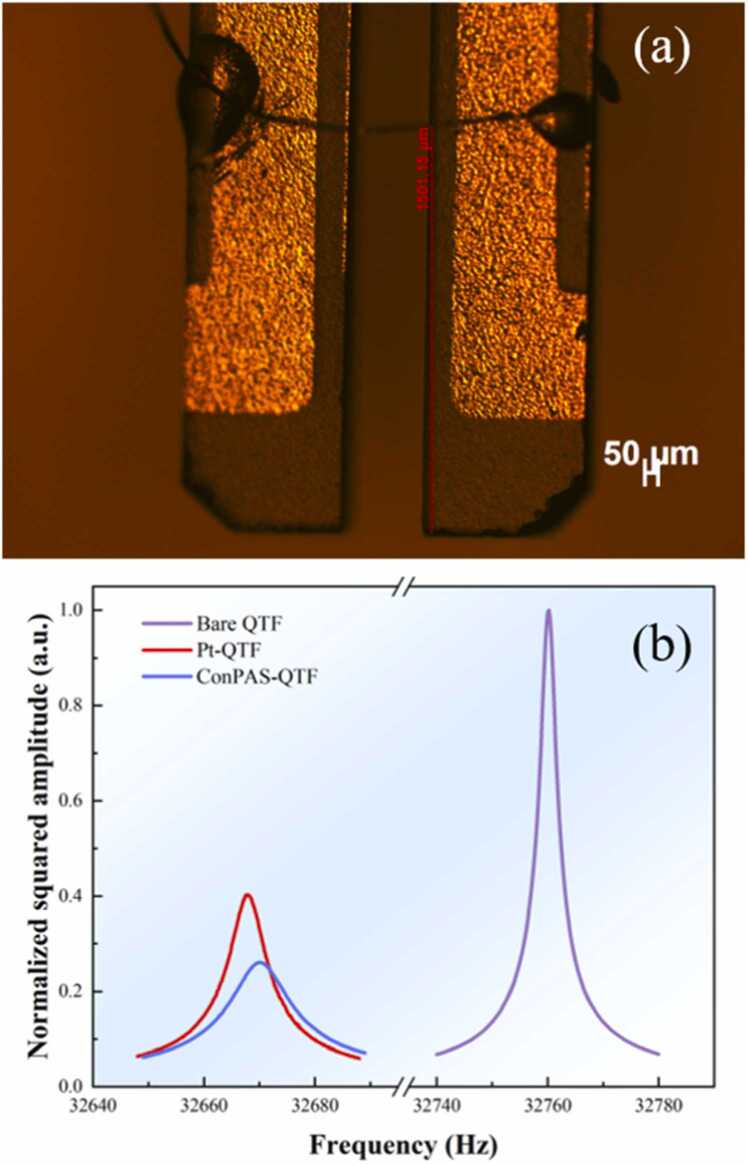
(a) Photograph of a Pt-QTF; (b) Conductance spectroscopy of a bare QTF, a Pt-QTF, and a ConPAS-QTF.

Fig. 2(b) assesses the influence of structural modifications on the resonance behavior of the QTF, frequency sweep measurements were conducted for three configurations: the bare QTF, the QTF modified with a platinum microwire, and the Pt-modified QTF further integrated with acoustic resonator tubes, named ConPAS-QTF. The bare QTF exhibited a sharp resonance peak at 32760.2 Hz with a high Q-factor of 11879, indicating minimal intrinsic damping. The photograph of a Pt-modified QTF was shown in the Fig. 2(a). A high-purity (99.99 %) platinum microwire with a diameter of 15 μm and a length of ∼2 mm was used. After bridging the prongs with a Pt microwire, the resonance frequency shifted downward to 32667.9 Hz due to the altered mechanical stiffness, while the Q-factor decreased to 5354. Upon further integration with a pair of stainless-steel acoustic resonator tubes, each 4.0 mm in length and 0.8 mm in outer diameter, symmetrically positioned along the optical axis, the resonance frequency was measured at 32670.1 Hz, and the Q-factor further declined to 3212 as a result of resonance coupling effects. Despite this reduction, the introduction of resonator tubes significantly enhanced the amplitude of the photoacoustic signal, confirming improved acoustic confinement and energy transfer efficiency. The resonance peak was extracted by applying Lorentzian curve fitting to the amplitude-frequency response. The quality factor was determined as the ratio of the resonance frequency *f*_0_ to the full width at half maximum *Δf*.

**Fig. 3:**
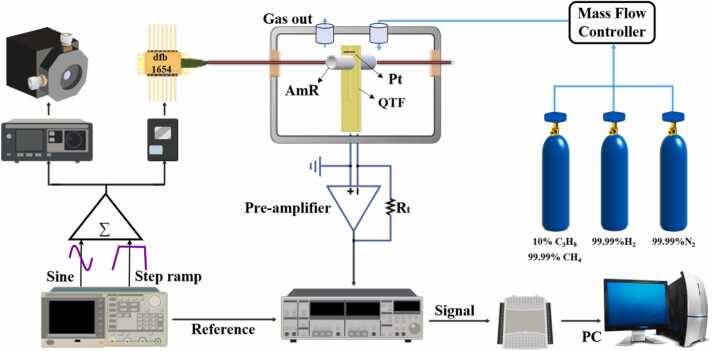
Schematic diagram of the ConPAS experimental setup.

A schematic of the experimental setup used for conductance–photoacoustic spectroscopy (ConPAS) measurements is shown in Fig. 3. An arbitrary waveform generator (Tektronix AFG3102) was employed to produce two modulation signals: a sinusoidal waveform for driving the laser modulation and a pulsed step-ramp waveform to induce the beat-frequency effect. These signals were superimposed and sent to the laser driver. For gas excitation, two lasers were used: an interband cascade laser (ICL) centered at 3370 nm for propane detection and a distributed feedback (DFB) diode laser centered at 1654 nm for methane detection. The laser beam was collimated and passed through a gas cell containing controlled mixtures of hydrogen, nitrogen, and either propane or methane, before being directed toward the ConPAS sensor. The output signal from the QTF was amplified using a custom transimpedance amplifier with a feedback resistance of 10 MΩ. To suppress electromagnetic interference, both the QEPAS sensor head and preamplifier were housed in a compact electromagnetic shielding enclosure. The amplified signal was subsequently demodulated at the first harmonic (1 *f*) using a lock-in amplifier (Stanford Research Systems SRS830) configured with a 1 ms time constant and a 12 dB/octave filter slope. The demodulated signal was digitized via a data acquisition card (National Instruments USB-6009) and recorded on a computer. Gas mixtures were prepared using high-purity cylinders of N₂ (99.999 %), H₂ (99.999 %), CH₄ (99.999 %), and 10 % C₃H₈. A gas dilution system equipped with three mass flow controllers (MFCs) was used to dynamically generate target compositions. To minimize noise and flow perturbation within the high-power optical module, the total gas flow rate was maintained at 200 standard cubic centimeters per minute (SCCM). The entire system was managed through a custom-developed LabVIEW program, and all experiments were conducted under ambient atmospheric pressure conditions.

**Fig. 4:**
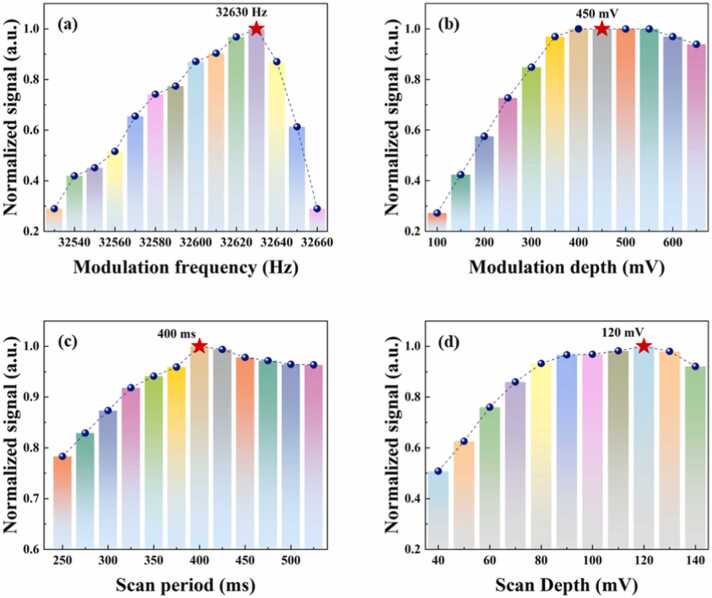
Optimization of modulation waveforms for photoacoustic signal generation in the ConPAS system. (a, b) Normalized signal amplitudes as functions of sinusoidal modulation frequency and depth, respectively (c, d) Effects of scan signal period and depth on normalized signal amplitude.

Two modulation waveforms, sinusoidal and step ramp, were systematically adjusted to drive the laser source for efficient signal generation. As ConPAS is a multidimensional signal system, parameters such as the modulation frequency and modulation depth, as well as the period and depth of the scanning signal, have all been systematically investigated, as shown in the Fig. 4. The modulation signal (sinusoidal waveform) was ultimately configured with a frequency of 32,630 Hz and a peak-to-peak amplitude of 450 mV, based on a series of parametric sweeps aimed at maximizing the signal-to-noise ratio while maintaining system stability, as illustrated in Figs. 4(a) and 4(b). This modulation frequency was deliberately selected to closely match the mechanical resonance of the modified quartz tuning fork (QTF), thereby ensuring efficient energy transfer and minimal phase mismatch between optical excitation and acoustic response. In parallel, the parameters of the scan signal (step ramp waveform), responsible for inducing the temporal beat effect, were optimized to a duration of 400 ms and a peak-to-peak voltage of 120 mV, as depicted in Figs. 4(c) and4(d). The step ramp facilitates quasi-continuous tuning of the laser injection current, enabling periodic scanning across the absorption features of the target gases (methane and propane). Specifically, the step-ramp signal consists of 3125 points in total, segmented into three regions: a rapid rising edge with 100 points, a constant holding region with 3000 points, and a fast falling edge with 25 points. The chosen ramp duration ensures sufficient temporal resolution for capturing the photoacoustic amplitude envelope associated with gas concentration changes.

## 3 Results and discussion

### 3.1 Assessment of ConPAS sensor performance

**Fig. 5:**
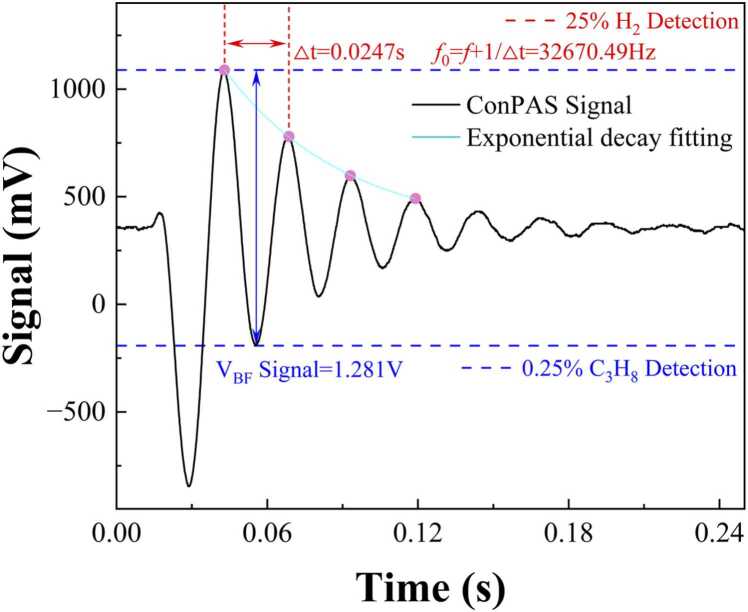
The signal of ConPAS for H_2_ and C_3_H_8_ mixture.

To generate the photoacoustic signal, the sinusoidal modulation is set at 32630 Hz, resulting in a difference of ∼40 Hz relative to the QTF’s resonance. The modulation amplitude was set to 22.5 mA, calculated from an optimized 450 mV driving signal (Section 2.2) and a modulation coefficient of 1 V per 500 mA, considering a 20 dB attenuator between the waveform generator and the laser driver. Concurrently, a pulsed harmonic signal sweeps the laser current from 90.1 mA to 92.1 mA at 20 ℃, encompassing the propane absorption line at 3369.74 nm. As shown in Fig. 5, take the C_3_H_8_/H_2_ detection as an example, the detected signal exhibits an exponential decay. By examining the periodicity in the horizontal direction (marked in red), we identify the QTF’s effective resonance, which shifts in response to hydrogen concentration. Calibration data then relates this frequency shift to the hydrogen concentration of 25 %. Meanwhile, the blue markers in the vertical direction track the amplitude of the beat signal peaks, which directly correlates with the propane absorption level. The difference between a peak and its subsequent trough quantifies the signal amplitude change, thereby yielding the propane concentration of 0.25 %. Thus, with a single measurement and a single output trace, the ConPAS approach simultaneously determines hydrogen and propane concentrations, showcasing its unique advantage in integrated, dual-species gas sensing.

### 3.2 C_3_H_8_, CH_4_ and H_2_ detection

**Fig. 6:**
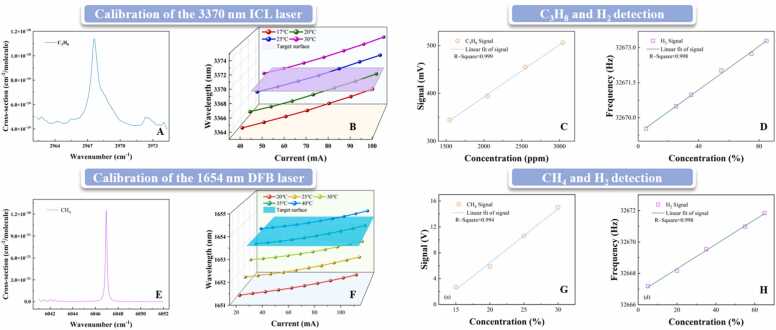
Spectroscopic and sensing characterization of the ConPAS system for propane–hydrogen and methane–hydrogen mixtures. (a)Absorption cross-section of C₃H₈ across the mid-infrared spectrum. (b)Calibration plot of the 3370 nm interband cascade laser showing emission wavelength versus drive current at various temperatures. (c)Photoacoustic signal amplitude versus C₃H₈ concentration. (d)QTF resonance frequency shift as a function of H_2_ concentration. (e) Absorption cross-section of CH₄ across the near-infrared spectrum. (f)Calibration plot of the 1.654 µm DFB laser showing emission wavelength versus drive current at various temperatures. (g)Photoacoustic signal amplitude versus CH₄ concentration. (h)QTF resonance frequency shift as a function of H_2_ concentration.

Because the HITRAN database does not provide line strength data for propane in the 2–4 μm spectral range, absorption cross-section data were used to identify the optimal absorption features[84]. The spectroscopic profile revealed a prominent absorption cross-section peak at 2967.6 cm⁻¹ , corresponding to a wavelength of 3369.735 nm, as shown in Fig. 6(a). The output of an interband cascade laser (ICL) was calibrated across various temperatures and injection currents. As illustrated in Fig. 6**(b)**, the laser wavelength precisely overlapped with the propane absorption peak at 20 ℃ and 91 mA, providing the operating parameters for subsequent measurements. Under a fixed hydrogen concentration of 50 %, the photoacoustic signal amplitude exhibited a strong linear increase with propane concentration from 500 ppm to 3000 ppm, yielding a regression coefficient of R² = 0.999, as presented in Fig. 6**(c)**. When the propane concentration was fixed at 2500 ppm and hydrogen was varied from 0 % to 100 %, the resonance frequency of the ConPAS-QTF increased linearly with hydrogen content, with a regression coefficient of R² = 0.998, as shown in Fig. 6**(d)**.

A parallel investigation was conducted for methane. HITRAN data revealed a significant absorption cross-section peak centered at 6047 cm⁻¹[85], corresponding to a wavelength of 1653.7 nm, as shown in Fig. 6(e). Calibration of the distributed feedback (DFB) laser diode revealed that the emission targeted the absorption line at 40 ℃ and 80 mA, as depicted in Fig. 6(f). When the hydrogen concentration was held constant at 5 %, increasing methane from 5 % to 30 % resulted in a near-linear increase in photoacoustic signal amplitude, with a regression coefficient of R² = 0.994, as shown in Fig. 6(g). Conversely, with methane fixed at 30 % and hydrogen varied from 0 % to 100 %, the resonance frequency of the ConPAS-QTF exhibited a linear increase, producing an R² of 0.998, as shown in Fig. 6(h). These results confirm the repeatability and analyte-specific selectivity of the ConPAS system for multicomponent gas sensing.

To evaluate the detection sensitivity of the ConPAS system, signal-to-noise ratio (SNR) and minimum detection limit (MDL) analyses were calculated. For the photoacoustic signal, the standard deviation of the baseline noise was measured to be 7.5 mV. At a propane concentration of 0.3 %, the signal amplitude reached 500 mV, yielding an SNR of 66.67 and a corresponding MDL of 45 ppm. For methane, a 30 % concentration produced a signal amplitude of 16 V, leading to an SNR of 2133.3 and an MDL of 140.6 ppm. For hydrogen, the frequency noise of the QTF was characterized by a standard deviation of 0.07 Hz. Based on the calibration slope obtained from the hydrogen concentration sweep (60 % change corresponding to a 5.92 Hz frequency shift), the MDL for hydrogen detection was calculated to be approximately 0.69 %.

## 4 Conclusions

In this work, we present a conductance–photoacoustic spectroscopy (ConPAS) technique for the simultaneous detection of hydrogen and hydrocarbon components using a single quartz tuning fork (QTF) sensor modified with a platinum microwire. The method integrates hydrogen-sensitive mechanical modulation and beat-frequency photoacoustic signal acquisition within a unified detection pathway. Hydrogen concentration is quantified via resonance frequency shifts induced by Pt–H₂ interactions, while the concentration of infrared-active hydrocarbons, such as propane and methane, is determined from the amplitude of the ConPAS signal. Quantitative analysis yields a minimum detection limit (MDL) of 0.69 % for hydrogen based on frequency resolution, and MDLs of 40.26 ppm for propane and 133.7 ppm for methane based on signal-to-noise ratio analysis. These results confirm that ConPAS enables decoupled, dual-species sensing using a single signal output and offers a compact, calibration-compatible solution for multi-component gas monitoring in hydrogen-containing environments.

Furthermore, the modular design allows the Pt microwire to be replaced with other chemically responsive materials, such as silkworm silk combined with a 2 μm laser for H₂O/CO₂ mixed gas analysis, thus extending the sensing capability to a broader class of gas mixtures. Sensor performance can be further improved with the incorporation of low-frequency custom QTFs in future developments, which will broaden the application spectrum of this sensing platform [86], [87], [88]. Overall, the architecture provides a scalable and flexible solution for real-time multi-component gas detection, leveraging the molecular selectivity of functional materials and the enhanced sensitivity of quartz-based spectroscopic techniques.

## CRediT authorship contribution statement

**Ruobin Zhuang:** Writing – original draft, Visualization, Software, Methodology, Investigation, Formal analysis, Data curation. **Jianfeng He:** Writing – review & editing, Supervision, Methodology, Conceptualization. **Haoyang Lin:** Supervision, Software. **Huijian Luo:** Supervision, Methodology. **Leqing Lin:** Validation, Investigation. **Lihao Wang:** Validation, Investigation. **Bin Liu:** Resources. **Wenguo Zhu:** Resources.**Yongchun Zhong:** Resources. **Jianhui Yu:** Resources. **Markus Sigrist:** Writing – review & editing, Formal analysis. **Huadan Zheng:** Writing – review & editing, Writing – original draft, Supervision, Project administration, Methodology, Formal analysis, Conceptualization.

## Declaration of Competing Interest

We wish to confirm that there are no known conflicts of interest associated with this publication and there has been no significant financial support for this work that could have influenced its outcome.

We confirm that the manuscript has been read and approved by all named authors and that there are no other persons who satisfied the criteria for authorship but are not listed. We further confirm that the order of authors listed in the manuscript has been approved by all of us.

We confirm that we have given due consideration to the protection of intellectual property associated with this work and that there are no impediments to publication, including the timing of publication, with respect to intellectual property. In so doing we confirm that we have followed the regulations of our institutions concerning intellectual property. We understand that the Corresponding Author is the sole contact for the Editorial process (including Editorial Manager and direct communications with the office). He/she is responsible for communicating with the other authors about progress, submissions of revisions and final approval of proofs.

## Data Availability

Data will be made available on request.
